# Physical activity and sedentary behavior patterns and sociodemographic correlates in 116,982 adults from six South American countries: the South American physical activity and sedentary behavior network (SAPASEN)

**DOI:** 10.1186/s12966-019-0839-9

**Published:** 2019-08-20

**Authors:** André O. Werneck, Se-Sergio Baldew, J. Jaime Miranda, Oscar Díaz Arnesto, Brendon Stubbs, Danilo R. Silva, on the behalf of the South American Physical Activity and Sedentary Behavior Network (SAPASEN) collaborators

**Affiliations:** 1 Department of Physical Education, Universidade Estadual Paulista “Júlio de Mesquita Filho”, Rua Roberto Símonsen, 305, 19060-900, Presidente Prudente, São Paulo Brazil; 2 Department of Physical Therapy, Faculty of Medical Sciences, Anton de Kom University of Suriname, Paramaribo, Suriname; 3 Facultad de Medicina “Alberto Hurtado”, Universidad Peruana Cayetano Heredia, Lima, Peru; 4 CRONICAS Centre of Excellence in Chronic Diseases, Universidad Peruana Cayetano Heredia, Lima, Peru; 5 Hospital Británico, Montevideo, Uruguay; 6 Department of Psychological Medicine, Institute of Psychiatry, Psychology and Neuroscience, King’s College London, De Crespigny Park, London, SE5 8AF UK; 7 South London and Maudsley NHS Foundation Trust, London, UK; 8 Department of Physical Education, Federal University of Sergipe – UFS, São Cristóvão, Brazil

**Keywords:** Sedentary lifestyle, Inequalities, Adult, Exercise

## Abstract

**Background:**

Physical inactivity and sedentary behavior are major concerns for public health. Although global initiatives have been successful in monitoring physical activity (PA) worldwide, there is no systematic action for the monitoring of correlates of these behaviors, especially in low- and middle-income countries. Here we describe the prevalence and distribution of PA domains and sitting time in population sub-groups of six south American countries.

**Methods:**

Data from the South American Physical Activity and Sedentary Behavior Network (SAPASEN) were used, which includes representative data from Argentina (*n* = 26,932), Brazil (*n* = 52,490), Chile (*n* = 3719), Ecuador (*n* = 19,851), Peru (*n* = 8820), and Suriname (*n* = 5170). Self-reported leisure time (≥150 min/week), (≥150 min/week), transport (≥10 min/week), and occupational PA total (≥10 min/week), as well as sitting time (≥4 h/day) were captured in each national survey. Sex, age, income, and educational status were exposures. Descriptive statistics and harmonized random effect meta-analyses were conducted.

**Results:**

The prevalence of PA during leisure (Argentina: 29.2% to Peru: 8.6%), transport (Peru: 69.7% to Ecuador: 8.8%), and occupation (Chile: 60.4 to Brazil 18.3%), and ≥4 h/day of sitting time (Peru: 78.8% to Brazil: 14.8%) differed widely between countries. Moreover, total PA ranged between 60.4% (Brazil) and 82.9% (Chile) among men, and between 49.4% (Ecuador) and 74.9% (Chile) among women. Women (low leisure and occupational PA) and those with a higher educational level (low transportation and occupational PA as well as high sitting time) were less active. Concerning total PA, men, young and middle-aged adults of high educational status (college or more) were, respectively, 47% [OR = 0.53 (95% CI = 0.36–0.78), I^2^ = 76.6%], 25% [OR = 0.75 (95% CI = 0.61-0.93), I^2^ = 30.4%] and 32% [OR = 0.68 (95% CI = 0.47-1.00), I^2^ = 80.3%] less likely to be active.

**Conclusions:**

PA and sitting time present great ranges and tend to vary across sex and educational status in South American countries. Country-specific exploration of trends and population-specific interventions may be warranted.

**Electronic supplementary material:**

The online version of this article (10.1186/s12966-019-0839-9) contains supplementary material, which is available to authorized users.

## Introduction

Engaging in regular physical activity (PA) and decreasing sedentary behavior are recognized as protective lifestyle behaviors against several non-communicable diseases, mental disorders, and all-cause mortality [1, 2]. Therefore, the World Health Organization (WHO) recommends that initiatives need to be taken on regional, national, and individual levels to stimulate PA and decrease sedentary behavior [3]. In order to develop effective interventions, it is important to detect trends in these lifestyle behaviors in an early stage and to identify their determinants [4]. This could be achieved through global monitoring of PA and sedentary behavior. The Global Observatory for Physical Activity -GoPA! [5] and the World Health Organization Working Group [3, 6] are global initiatives for PA monitoring. These initiatives initially focused on PA but not on sedentary behavior and their combination. Recently, *GoPA!* started work toward the inclusion of sedentary behavior in the report cards (which describe several PA indicators of each country, including prevalence, research indicators, and PA policies), however, this is still in the implementation phase.

Previous transnational studies, such as the World Health Survey, that assessed PA specifically among low- and middle-income countries primarily focused on the association between socioeconomic indicators and, especially, the leisure time and occupational domain of PA [7, 8]. These studies reported a positive relationship between socioeconomic status (particularly educational status) and leisure-time PA [9–11] but a negative relationship between socioeconomic status and occupational PA [10, 11]. However, there is still no clear understanding of the association of socioeconomic status with other domains of PA, overall PA and sedentary behavior, from, especially, middle-income countries. Moreover, sex/age group differences for these two behaviors have also not been widely studied. Furthermore, there is still a need for studies aimed at the relation with sedentary behavior from low and middle -income countries.

In South America, there have been initiatives that promote PA through campaigns and environmental strategies, such as the RAFA/PANA, AGITA program and GUIA project [12, 13]. However, to the best of our knowledge, there is no systematic empirical monitoring at regional levels or correlates/determinants of PA and sedentary behavior, which could contribute to the development and evaluation of effective interventions considering local specificities. The latter is of great importance, since the South American continent represents 12% of world’s surface and 6% of the global population, with a range of cultural differences and large variation in the distribution of diseases and lifestyle behaviors. Furthermore, the continent underwent an accelerated urbanization process and is characterized by recent aging of the population and considerable levels of poverty [14–17].

In order to counter this, the South American Physical Activity and Sedentary Behavior Network (SAPASEN) was established in 2018 with the aim of monitoring the specific prevalence and associated factors of PA and sedentary behavior in South America, using national representative datasets of each country. This study aims to describe the PA prevalence within the different PA domains and sedentary behavior as well as distribution according to sociodemographic characteristics. In addition, we conducted a harmonized meta-analysis according to each behavioral domain in order to better understand correlates of these behaviors in South American adults.

## Methods

### Design

SAPASEN was formed by a representative body of researchers and policy makers from South American countries through an effort to jointly examine empirical data available from the continent. Firstly, the network targeted at least one representative of each country. Researchers were invited based on the productivity and representativeness of PA in each country [18]. Six of the ten national representative datasets available were used in this first analysis (Argentina, Brazil, Chile, Ecuador, Peru, Suriname). Paraguay, Uruguay and Venezuela did not provide data and Colombia did not reply to the invitation to be part of SAPASEN. Therefore, in this study, we present data from six nationally representative studies conducted among adults (18-64y).

### Sample

We used open data from Argentina (Encuesta Nacional de Factores de Riesgo 2013), Brazil (Pesquisa Nacional de Saúde 2013), Chile (Encuesta Nacional de Salud 2009–2010), Ecuador (Encuesta Nacional de Salud y Nutrición 2012), Peru (Encuesta Nacional de Hogares, Módulo de Mediciones Antropométricas, 2011), and Suriname (The Suriname Health Study, 2013). Data from each country were pooled, excluding participants younger than 18y and older than 64y. This was different only in Ecuador’s dataset, which included adults between 18y and 59y. All samples were calculated through complex sampling. The common primary sample units were the census units of each country. More details on the sampling methodology can be found in the report of each country [19–24]. After the exclusion of subjects older than 64y and younger than 18y as well as missing data (including exposures and outcomes), a final sample of 116,982 adults (Argentina = 26,932 (from 26,989 within age range); Brazil = 52,490 (from 52,490 within age range); Chile = 3719 (from 4056 within age range); Ecuador = 19,851 (from 19,883 within age range); Peru = 8820 (from12,733 within age range with PA data); and Suriname = 5170 (from 5404 within age range) was used for the analysis. Sampling weights were used in each study.

### Physical activity and sedentary behavior

To assess PA and sedentary behavior, the International Physical Activity Questionnaire (IPAQ) [25] was used in Argentina, Ecuador, and Peru and the Global Physical Activity Questionnaire (GPAQ) [26] in Chile and Suriname. Brazil used a specific questionnaire, which was an adaptation of the GPAQ. Even though all questionnaires included questions regarding each PA domain (leisure time, transportation, and occupational) and total sitting time, the surveys from Argentina and Ecuador did not include the occupational PA domain, whereas Ecuador and Brazil did not include sitting time. In addition, aiming to improve harmonization, we did not include the household domain, which forms part of the IPAQ. On the other hand, the Brazilian survey included total TV-viewing, which was only used for descriptive analyses. There were some differences between the questionnaires, the main difference being that the IPAQ refers to the last 7 days and the GPAQ to a typical week. We adopted the cut-off points of 150 min of moderate to vigorous PA per week for leisure-time PA and total PA (sum of PA domains), according to WHO recommendations [27], and at least 10 min/ day of occupational and transport PA. Given that there are no specific recommendations for these last domains, our aim was to screen for individuals who practice at least one minimum bout of PA as described in questionnaires such as the IPAQ [25]. Moreover, we adopted ≥4 h/day as a cut-off point for sitting time, which is a critical point for several negative outcomes, including cardiovascular diseases, mental disorders, and all-cause mortality and has been widely used in previous research [28–31].

### Sociodemographic characteristics

Sex, chronological age (18-34y, 35-49y, 50-64y), level of education, and income were considered as sociodemographic indicators/characteristics for descriptive analysis. For the level of education, we formed four categories based on the final completed level of formal education: a) no formal education, b) less than secondary, c) secondary, and d) college or more. For the harmonized meta-analysis, we collapsed groups “a” and “b” to compare against groups “c” and “d”. In the meta-analysis, we included the results of “d” against “a” and “b”, given that our aim was to compare those with lower education against those with higher education. Finally, the minimum wage of each country (except Ecuador and Peru owing to the absence of data) was used to categorize participants into income level, comparing individuals who earn more than one minimum wage with those who earn less.

### Statistics

Percentage and 95% confidence intervals were used to describe the prevalence of each outcome and to compare groups [32]. For the harmonizing process, logistic regression models were used in each study, with sex (women vs. men) and educational status (college or more vs. lower than secondary school) as main exposures. We stratified analyses of total PA by sex considering consistent differences between sexes in global estimates [33]. Sampling weights were used for all analyses. Subsequently, random effect meta-analyses for logistic parameters were conducted, using the command “metan” of STATA. To assess the level of heterogeneity between studies, the Higgin’s I2 statistic [34] was calculated based on country-wise estimates, which represents the heterogeneity that is not explained by sampling error. The following cut-off values were adopted: < 40: low, between 41 and 60: moderate, and > 60: high [35]. All analyses were conducted using STATA 15.1 software.

## Results

From the initial sample, 116,982 adults from six countries provided complete data. In Table 1, it is clear that in all countries there was equal distribution of men and women, as well as age groups. Suriname presented the highest percentage of participants with no formal education, Chile the highest percentage of participants within the minimum wage level of income, and Argentina the lowest percentage of participants with no formal education and within the minimum wage level of income. Peru presented the lowest rate of leisure time PA, but showed the highest prevalence for transport PA, followed by Chile and Argentina. Peru also presented the highest prevalence of ≥4 h/day of sitting time.

**Table 1:** Characteristics of sample by country

	Country					
	Argentina (*n* = 26,932)	Brazil (*n* = 52,490)	Chile (*n* = 3719)	Ecuador (*n* = 19,851)	Peru (*n* = 8820)	Suriname (*n* = 5170)
Sex						
Men	48.7 (47.6 to 49.9)	47.6 (46.8 to 48.6)	48.9 (46.3 to 51.6)	48.2 (47.3 to 49.1)	48.0 (46.7 to 49.4)	49.1 (47.5 to 50.7)
Women	51.3 (50.1 to 52.4)	52.4 (51.7 to 53.2)	51.1 (48.4 to 53.7)	51.8 (50.9 to 52.7)	52.0 (50.6 to 53.3)	50.9 (49.3 to 52.5)
Age group						
18-34y	45.3 (44.1 to 46.5)	42.8 (42.1 to 43.6)	38.6 (36.0 to 41.2)	51.8 (50.5 to 53.1)	43.2 (41.9 to 44.6)	44.6 (42.9 to 46.2)
35-49y	31.3 (30.3 to 32.4)	32.2 (31.5 to 32.9)	37.1 (34.6 to 39.7)	33.2 (32.0 to 34.5)	33.9 (32.6 to 35.1)	34.2 (32.7 to 35.7)
50-64y	23.4 (22.4 to 24.3)	25.0 (24.4 to 25.7)	24.3 (22.3 to 26.5)	15.0 (13.8 to 16.3)	22.9 (21.8 to 24.1)	21.2 (20.1 to 22.5)
Educational status						
No education	0.9 (0.7 to 1.1)	4.2 (3.9 to 4.5)	1.0 (0.6 to 1.4)	1.4 (1.1 to 1.7)	4.1 (3.7 to 4.6)	7.8 (7.0 to 8.6)
Less than secondary	43.5 (42.3 to 44.6)	40.2 (39.4 to 40.9)	31.6 (29.2 to 34.0)	50.0 (48.0 to 52.0)	37.3 (36.0 to 38.5)	66.9 (65.3 to 68.5)
Secondary education	39.3 (38.1 to 40.4)	40.4 (39.7 to 41.2)	58.7 (56.1 to 61.2)	29.8 (28.6 to 31.0)	39.4 (38.1 to 40.8)	18.1 (16.8 to 19.5)
College or more	16.4 (15.6 to 17.2)	15.2 (14.6 to 5.7)	8.9 (7.3 to 10.7)	18.8 (17.2 to 20.5)	19.2 (18.0 to 20.4)	7.3 (6.4 to 8.2)
Wage						
Minimum wage	21.3 (20.4 to 22.2)	17.8 (17.1 to 18.5)	28.1 (25.9 to 30.4)	–	–	25.3 (23.4 to 27.4)
More than minimum	78.7 (77.8 to 79.6)	82.2 (81.5 to 82.9)	71.9 (69.6 to 74.1)	–	–	74.7 (72.6 to 76.6)
Total PA (%)	60.2 (59.0 to 61.3)	55.4 (54.7 to 56.2)	79.2 (77.1 to 81.1)	58.2 (56.9 to 59.5)	70.0 (68.6 to 71.2)	61.3 (59.7 to 62.9)
Leisure time PA (%)	29.2 (28.2 to 30.3)	20.3 (19.7 to 21.0)	20.8 (18.7 to 23.0)	15.3 (14.4 to 16.4)	8.6 (7.9 to 9.4)	17.4 (16.1 to 18.8)
Transport PA (%)	63.6 (62.3 to 64.9)	51.3 (50.6 to 52.1)	66.2 (63.7 to 68.7)	8.8 (8.0 to 9.6)	69.7 (68.4 to 71.0)	27.5 (26.1 to 29.0)
Occupational PA	–	18.3 (17.7 to 18.9)	60.4 (57.8 to 62.9)	–	51.2 (49.9 to 52.6)	51.8 (50.2 to 53.4)
High sitting time (%)	58.4 (57.3 to 59.6)	14.8 (14.2 to 15.3)	35.5 (32.9 to 38.1)	–	78.8 (77.7 to 79.9)	53.0 (51.4 to 54.6)

Note. Values are presented in percentage and 95% confidence intervals. *Y* Years. Cut-off points for each physical activity domain were: Leisure time (≥150 min/week), transport (≥10 min/week), and occupational PA (≥10 min/week), and sitting time (≥4 h/day)

The prevalence of leisure time PA was higher among men than women in all countries except Argentina (Additional file 4: Table S1). Among men, Argentina, Chile, and Suriname presented the highest rates of leisure time PA (between 25 and 29%), while among women only Argentina presented a prevalence of leisure time PA between 25 and 29%. Furthermore, regarding the age groups in each country, the prevalence of leisure time PA was lower in older adults and with respect to educational level, it was higher in subjects with a higher educational status.

Transport PA was not consistently different among sexes or age groups, except in Brazil and Ecuador where the prevalence was higher among men. For educational level, the prevalence of transport PA was lower among participants with a higher educational status (college or more) in all countries. Occupational PA was higher in men, and lower in participants with a higher educational status. In addition, occupational PA was lower among older participants.

No differences were observed between sexes concerning sitting time, with the exception of Brazil. More than half of the Argentineans and Peruvians reported ≥4 h of sitting per day. Older subjects reported a lower prevalence of daily sitting, while subjects with a higher educational status presented a higher prevalence of sitting, with the exception of Brazil (Additional file 4: Table S1).

**Fig. 1:**
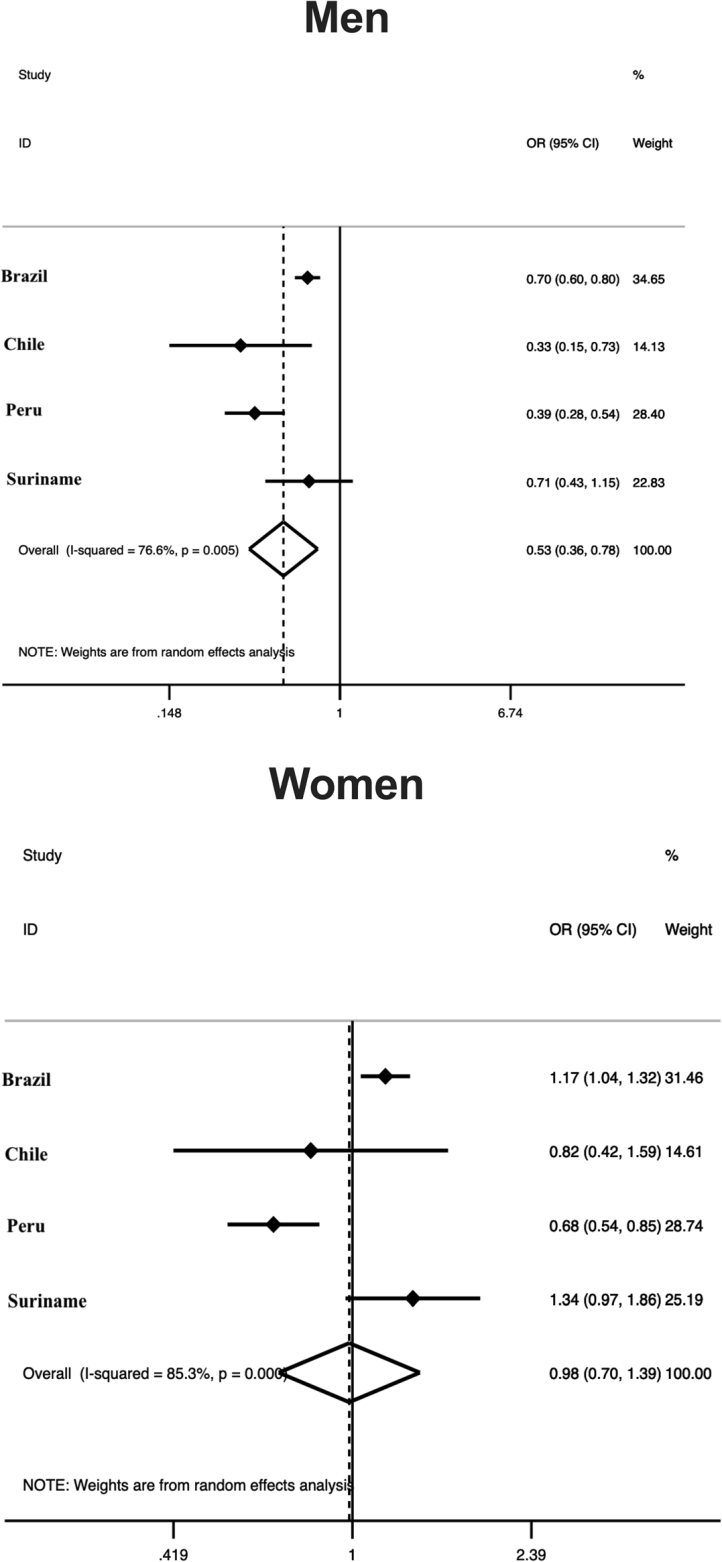
Harmonized meta-analysis of the association between total physical activity and educational status by sex. Odds ratio of educational status refers to college or more vs. lower than secondary school. Odds ratio results are adjusted by age group and calculated using sampling weights. Weights are from random effects analysis. OR, odds ratio. 95%CI, 95% confidence interval

**Fig. 2:**
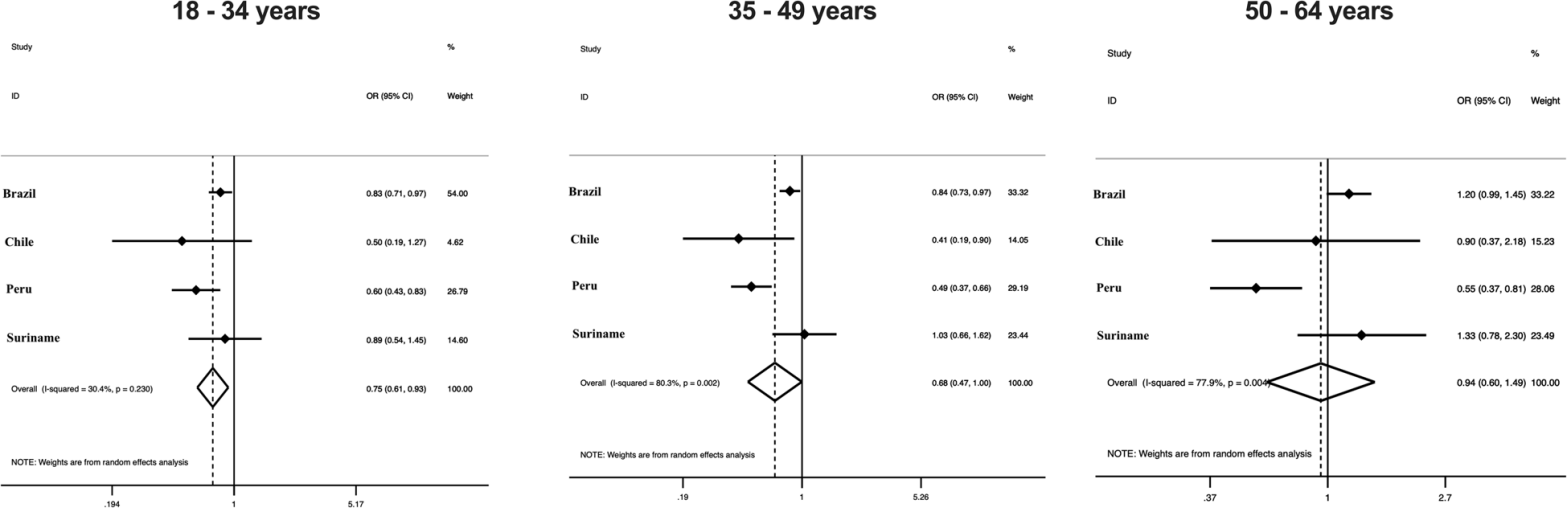
Harmonized meta-analysis of the association between total physical activity and educational status by age group. Odds ratio of educational status refers to college or more vs. lower than secondary school. Odds ratio results are adjusted by sex and calculated using sampling weights. Weights are from random effects analysis. OR, odds ratio. 95%CI, 95% confidence interval

The harmonized meta-analysis of the association between educational status and total PA according to sex is presented in Fig. 1. Men with a higher educational status were 47% [OR = 0.53 (95% CI = 0.36–0.78), I^2^ = 76.6%] less likely to be physically active compared to subjects with schooling lower than secondary school, while this association was not significant among women. The harmonized meta-analysis of the association between educational status and total PA according to age group is presented in Fig. 2. Participants with a higher educational status were less likely to be physically active compared to subjects with schooling lower than secondary school among young participants [25%-OR = 0.75 (95% CI = 0.61-0.93), I^2^ = 30.4%] and middle-aged adults [32%-OR = 0.68 (95% CI = 0.47-1.00), I^2^ = 80.3%]. On the other hand, this association was not consistent among older adults.

**Fig. 3:**
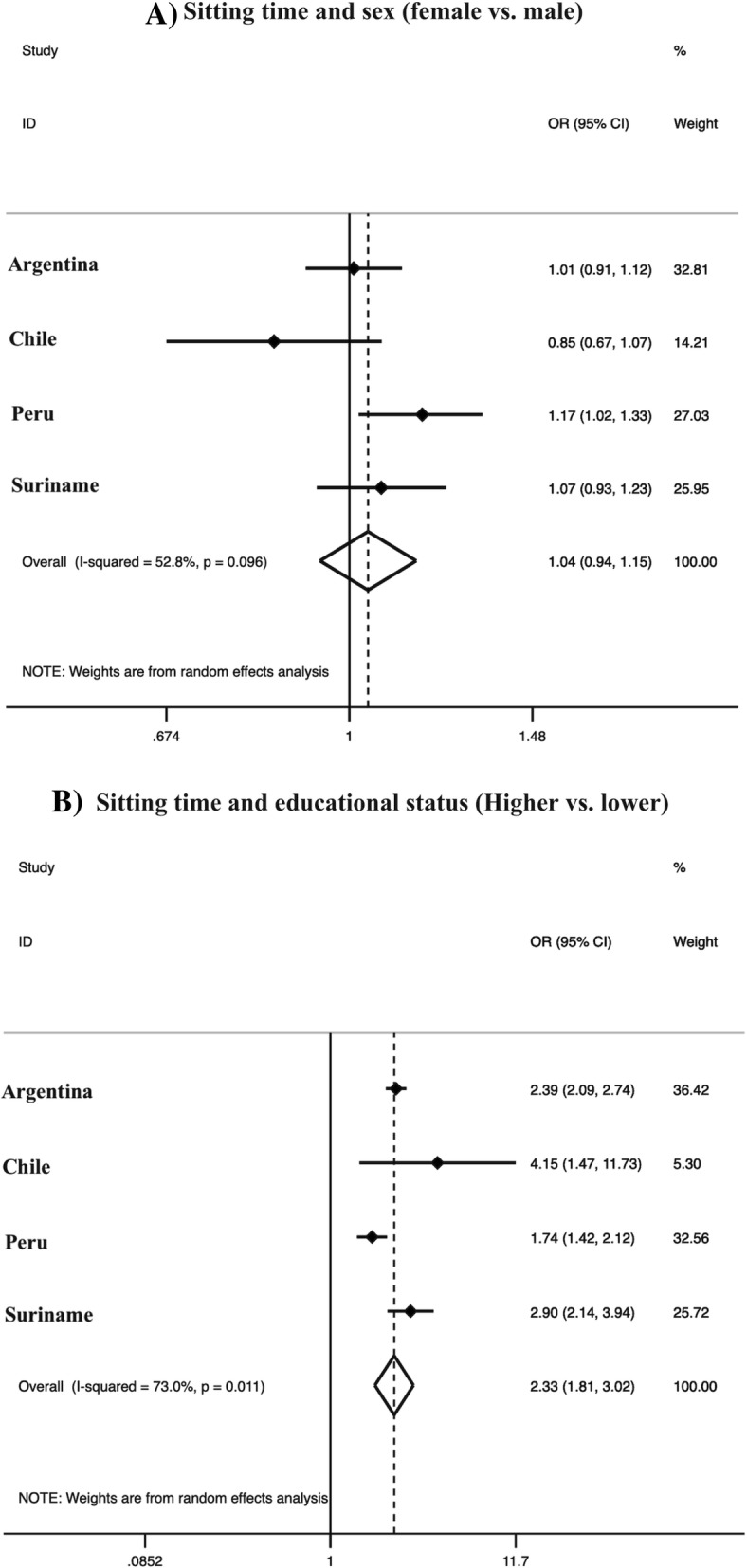
Harmonized meta-analysis of the association between sitting time (≥4 h/day) activity and sex/educational status. **a** Odds ratio of sex refers to women compared with men. **b** Odds ratio of educational status refers to college or more vs. lower than secondary school. Odds ratio results are adjusted by age group and leisure-time physical activity and calculated using sampling weights. Weights are from random effects analysis. OR, odds ratio. 95%CI, 95% confidence interval

For sitting time analysis, Brazil (only data on TV viewing) and Ecuador (without data) were not included. Sex was not associated with sitting time, while subjects with a higher educational status presented 133% [OR = 2.33 (95% CI = 1.81–3.02), I^2^ = 73.0] higher odds for more than 4 h/day of sitting (Fig. 3).

The harmonized meta-analyses of the association of PA domains with sex and educational status are presented in Additional file 1: Figure S1, Additional file 2: Figure S2, and Additional file 3: Figure S3. Despite the heterogeneous results, women showed 53% [OR = 0.47 (95% CI = 0.30–0.73), I^2^ = 98.4%] lower odds for undertaking more than 150 min of leisure time PA than men. Higher educational status was associated with 98% [OR = 1.98 (95% CI = 1.40–2.82), I^2^ = 92.3%] higher odds for presenting more than 150 min of leisure time PA per week when compared with the less than secondary school group (Additional file 1: Figure S1). On the other hand, sex was not associated with transport PA, while higher educational status was associated with lower odds for the transportation domain (43% less) [OR = 0.57 (95% CI = 0.54–0.61), I^2^ = 0.0%] (Additional file 2: Figure S2).

For occupational PA analysis, Argentina and Ecuador were not included due to the lack of data. Sex was also associated with occupational PA, in which women presented odds 52% [OR = 0.48 (95% CI = 0.29 = 0.80), I^2^ = 98.5] lower than men. Subjects with a higher educational status showed odds 55% [OR = 0.45 (95% CI = 0.26–0.76), I^2^ = 95.6] lower than subjects with less education for occupational PA (Additional file 3: Figure S3).

## Discussion

The findings presented in this article arise from a collaborative network aiming to monitor PA and sedentary behavior in South America and describes the relation of different PA domains and sitting time with country and sociodemographic characteristics. Other global initiatives like the World Health Survey, which aimed at behavior surveillance, only included Brazil, Uruguay, Ecuador, and Paraguay from South America [36], whereas the Global Physical Activity Observatory [5] focused in monitoring PA, and did not explore the association of sociodemographic factors with different domains of PA. Furthermore, there are no multinational surveys that consider sedentary behavior as a new public health issue. Therefore, the SAPASEN initiative has a pioneering role in providing scientific information on monitoring PA and sedentary behavior and their region-specific correlates and determinants.

In the current study, we analyzed the prevalence of different PA domains as well as sitting time according to country and sociodemographic characteristics. Although using different harmonization methods, the prevalence values found here were quite similar to a recent global systematic review [6]. We observed that men were more active in leisure time and occupational domains, while women were more active in transportation. Lower educational status was associated with a higher activity pattern, with the exception of leisure time PA. Moreover, greater educational status was associated with lower PA among men and younger adults, but not women, or middle-aged or older adults. These findings on the association of PA with sex and educational status corroborate the results from other low- and middle-income countries [33]. Therefore, our findings highlight the importance of difference in PA behavior between men and women and the role of educational status. For the association between PA and age groups, our findings are not consistent with previous studies. This could be explained by the difference in age range and age groups used in the studies.

Besides supporting previous evidence, this study from SAPASEN brings new insights on the association between sociodemographic characteristics and different domains of PA and sitting time in South America. Leisure time PA was greater among men, which could be due to several biological and cultural factors, as well as preferences for types of activities [37]. It is well recognized that the hormonal environment and body composition differences between men and women affect active behaviors. In addition, the social role of women in many cultures is associated with less active behaviors. The only exception here was Argentina, which reported similar rates of PA practice between sexes. Given the benefits of leisure time PA, which has strong associations with a reduced risk of multiple chronic diseases [1, 2], policies are needed to stimulate leisure time PA among women, especially in Ecuador and Peru, which were the countries that demonstrated the greatest inequalities concerning sex differences in this domain.

It is possible that higher levels of occupational PA can compensate the lower leisure-time PA of participants with lower educational status among men in South America. However, as women present lower occupational PA [38], this tends to occur only in men. Furthermore, younger adults are more likely to perform active work than older adults [39], which could explain the negative association with educational status.

This multinational study also reinforces the importance of socioeconomic status, i.e. educational status, as a correlate with PA practice [4]. Previous research from high-income countries has been equivocal when considering the association between socioeconomic status and PA [11, 40–42]. Based on their systematic review, Gidlow et al. [41] reported that in 24 studies there was a negative association between PA and educational level, while in 17 studies there was a positive association. However, Gidlow et al. included studies only on leisure time PA and studies on total PA. More recently, two systematic reviews, conducted almost exclusively among high-income countries [42, 43], found inconsistent evidence for the association between educational status and total PA.

The association between socioeconomic factors and PA becomes stronger when looking at the separate domains [11, 42]. There have been reports of stronger associations between socioeconomic condition and levels of leisure-time PA in middle-income countries [9, 11]. A higher socioeconomic position is associated with greater opportunities to practice leisure time PA, through a more favorable neighborhood as well as greater access to PA facilities [8]. On the other hand, a lower socioeconomic condition is consistently associated with higher occupational PA, even in high-income countries [10, 11]. For occupational PA there have been reports of a positive association as well as a negative association with socioeconomic factors. Higher socioeconomic status was associated with higher walkability access, which is associated with greater transport PA, whereas subjects with lower socioeconomic status have lower access to individual transport items, especially in middle-income countries, which is also associated with higher transport PA [8].

We found that Chile was the only country in which leisure PA was not associated with educational status. Chile has the greatest Human Development Index of South America and, consequently, lower inequality. Interestingly, the whole approach of the different domains of PA should help governments to indentify very inactive population subgroups and potential factors that influence this inactivity for the three domains. Hence, decisions can be taken to build places appropriate for leisure time PA such as parks, outdoor courts [8], and improved walkability of streets [33], and bicycle paths. It is important to highlight that these actions are included in national PA policies in Argentina [44], Brazil [45], Chile [46], Ecuador [47], and Suriname [48].

Concerning sitting time, subjects with a greater educational status presented higher odds for ≥4 h/day of sitting time. This result may be explained by the relationship between educational status and work characteristics. People with a higher educational status are more likely to have sedentary jobs, e.g. blue vs white-collar jobs. This finding confirms occupational PA results, which are inverse, with a higher educational status being associated with lower PA. Interestingly, in Brazil, we found the opposite using TV viewing as a proxy for sedentary behavior, in which subjects with greater educational status presented lower TV viewing. Considering the sum of daily domains analyzed, people with a higher educational status tend to be less active at work and more active during leisure time. Thus, the differences between the proxies of sedentary behavior can be explained by the fact the majority of TV viewing time occurs during leisure time. This reinforces the need to assess different domains and manifestations of sedentary behaviors as distinct outcomes.

Considering the negative effect of sedentary behaviors on health outcomes [2], there is a need to monitor these behaviors in national health surveys. However, up to now, there has been no international effort aiming to survey sedentary behavior worldwide. The greatest effort was the Study of AGEing and adult health (SAGE), which did not include any South American countries and focused on the older adult population [49]. The importance of strategies aiming to reduce sitting time should also be inserted in national policies. Currently, there are no strategies that aim to reduce sedentary behavior in South America. Uruguay considered including this topic in their national plan [50], but, to date, no interventions have been presented.

Another aim of SAPASEN is to standardize the assessment instruments for PA and sedentary behavior. In the national health surveys that we used, Chile and Suriname used the GPAQ questionnaire [26], while Argentina, Ecuador, and Peru used the IPAQ questionnaire [25]. Brazil used a specific questionnaire developed for the national health survey. The indicator of sitting time was the same for all surveys, except for Brazil, in which the only indicator of sitting time concerned TV viewing. Although indicatives suggest that some of these instruments provide similar estimates [51], the compatibility between surveys could be improved with standardization. This is the next challenge for the SAPASEN initiative [18].

The current study presents some limitations. Firstly, even though the aim of the SAPASEN is to build a representative dataset from each South American country, two countries reported the absence of national representative datasets on PA and sedentary behavior indicators after 2005 (Guyana and Bolivia). In addition, four countries did not make the data from their surveys available (Colombia, Paraguay, Uruguay and Venezuela). Despite these difficulties, we presented representative data covering a region with more than 320 million people, which covers 76% of the South American population. Although we used the most recent representative sample of the countries, data ranged from 2009 to 2014. However, recent global analysis found no temporal trend in physical inactivity between 2009 and 2015 [6]. Moreover, estimates derived from harmonized meta-analyses should be extrapolated with caution, considering that the questionnaires were different. A final limitation is that our measures of PA and sedentary behavior were based on self-report measures. Whilst this method enables the collection of data from large numbers of nationally representative data, added to which, the questionnaires have been validated, the method is prone to recall bias.

In conclusion, PA and sedentary behavior outcomes present great ranges and tend to vary according to sex and educational status in South American countries. Leisure time (men and high educational status), transportation (women and low educational status), occupational PA (men and low educational status), and total PA (men and low educational status), as well as high sitting time (high educational status) are more prevalent in specific population sub-groups. This first set of analyzes from SAPASEN provides information about inactive and sedentary groups that should receive attention from public health policies. Future studies in South America should explore modifiable correlates of these health behaviors in order to develop intervention strategies of health promotion in specific contexts.

## Additional files

Additional file 1: **Figure S1.** Harmonized meta-analysis of the association between leisure-time physical activity (≥150 min/week) and sex/educational status. A) Odds ratio of sex refers to women compared with men. B) Odds ratio of educational status refers to college or more vs. lower than secondary school. Odds ratio results are adjusted by age group and sitting time and calculated using sampling weights. (TIFF 431 kb)

Additional file 2: **Figure S2.** Harmonized meta-analysis of the association between transport physical activity (≥10 min/week) and sex/educational status. A) Odds ratio of sex refers to women compared with men. B) Odds ratio of educational status refers to college or more vs. lower than secondary school. Odds ratio results are adjusted by age group and sitting time and calculated using sampling weights. (TIFF 407 kb)

Additional file 3: **Figure S3.** Harmonized meta-analysis of the association between occupational physical activity (≥10 min/week) and sex/educational status. A) Odds ratio of sex refers to women compared with men. B) Odds ratio of educational status refers to college or more vs. lower than secondary school. Odds ratio results are adjusted by age group and sitting time and calculated using sampling weights. (TIFF 380 kb)

Additional file 4: **Table S1.** Prevalence of physical activity per domain and sitting time among South American countries by sociodemographic characteristics. (DOC 84 kb)

## Data Availability

All datasets are available on each governmental website, except data from Suriname.

## Abbreviations

CI: Confidence interval
GoPA!: Global Observatory for Physical Activity
OR: Odds ratio
PA: Physical activity
SAPASEN: South American physical activity and sedentary behavior network
