# Supplementary figures and images for: Physical activity and sedentary behavior patterns and sociodemographic correlates in 116,982 adults from six South American countries: the South American physical activity and sedentary behavior network (SAPASEN)
Source: Int J Behav Nutr Phys Act. 2019 Aug 20;16:68. doi: 10.1186/s12966-019-0839-9 (PMC6701122; doi:10.1186/s12966-019-0839-9)

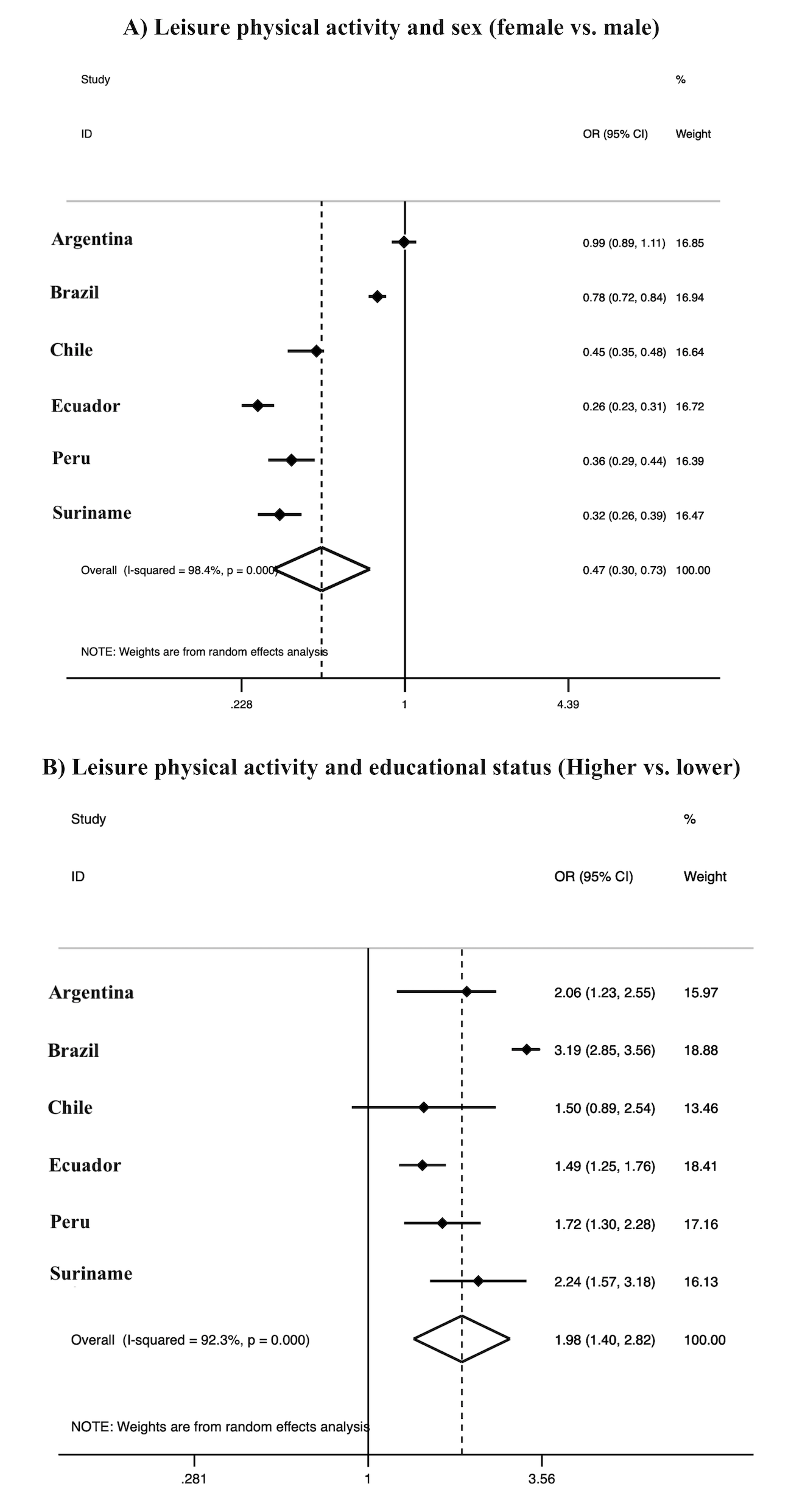

Supplement: Supplementary file 1 — Figure S1. Harmonized meta-analysis of the association between leisure-time physical activity (≥150 min/week) and sex/educational status. A) Odds ratio of sex refers to women compared with men. B) Odds ratio of educational status refers to college or more vs. lower than secondary school. Odds ratio results are adjusted by age group and sitting time and calculated using sampling weights. (TIFF 431 kb) [file 12966_2019_839_MOESM1_ESM.tiff]

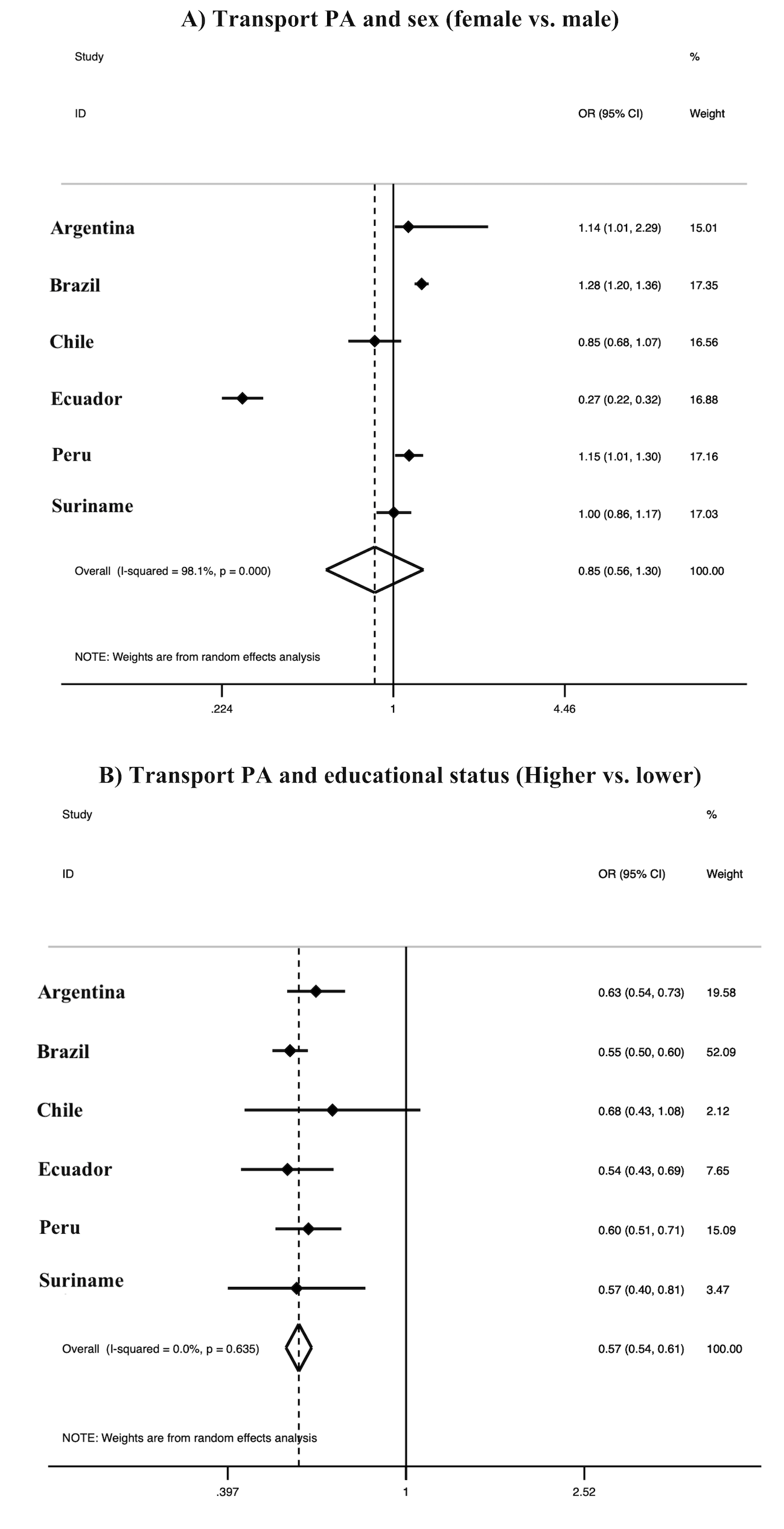

Supplement: Supplementary file 2 — Figure S2. Harmonized meta-analysis of the association between transport physical activity (≥10 min/week) and sex/educational status. A) Odds ratio of sex refers to women compared with men. B) Odds ratio of educational status refers to college or more vs. lower than secondary school. Odds ratio results are adjusted by age group and sitting time and calculated using sampling weights. (TIFF 407 kb) [file 12966_2019_839_MOESM2_ESM.tiff]

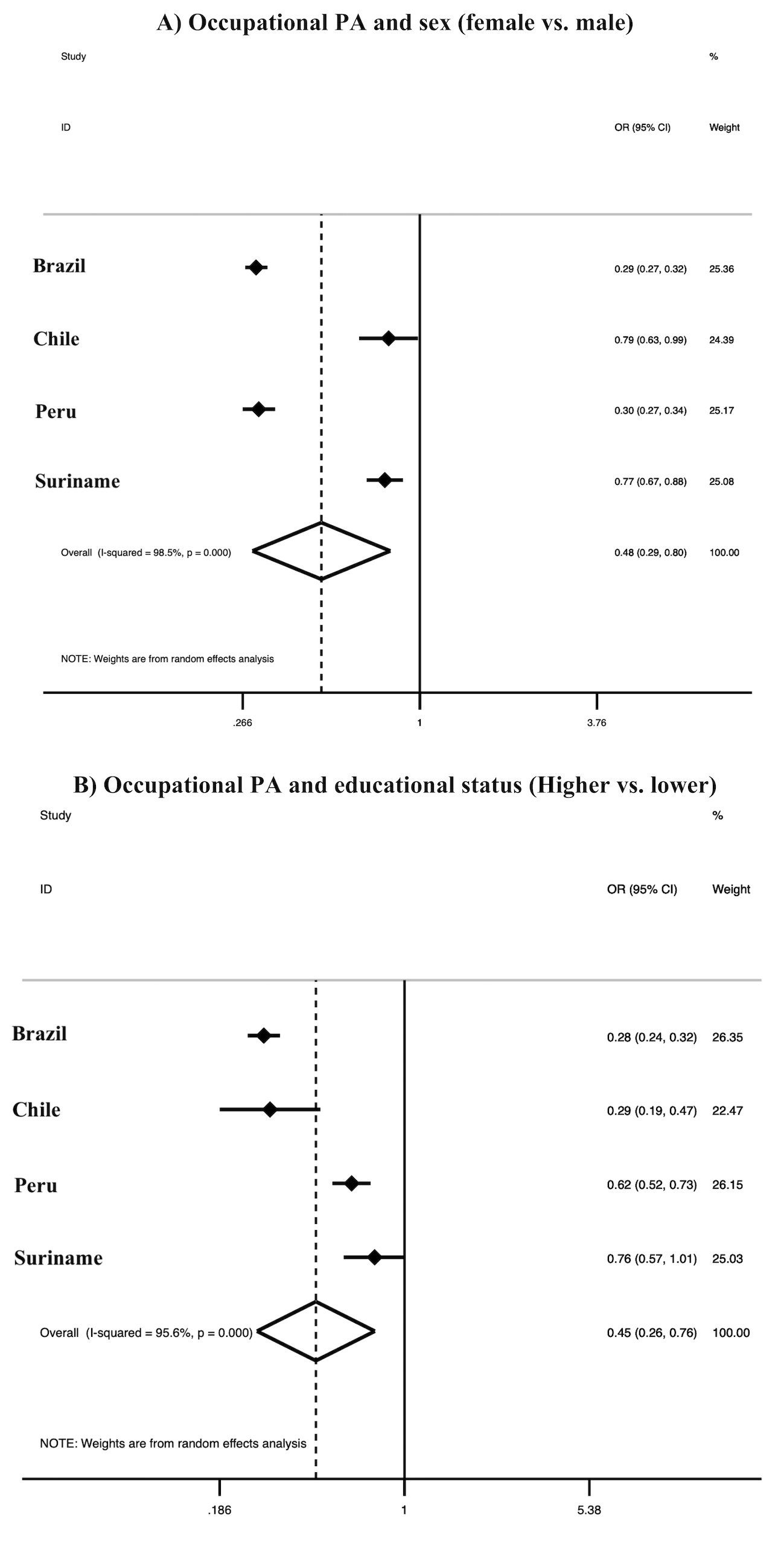

Supplement: Supplementary file 3 — Figure S3. Harmonized meta-analysis of the association between occupational physical activity (≥10 min/week) and sex/educational status. A) Odds ratio of sex refers to women compared with men. B) Odds ratio of educational status refers to college or more vs. lower than secondary school. Odds ratio results are adjusted by age group and sitting time and calculated using sampling weights. (TIFF 380 kb) [file 12966_2019_839_MOESM3_ESM.tiff]
