# Supplementary material for: Physical activity and sedentary behavior patterns and sociodemographic correlates in 116,982 adults from six South American countries: the South American physical activity and sedentary behavior network (SAPASEN)
Source: Int J Behav Nutr Phys Act. 2019 Aug 20;16:68. doi: 10.1186/s12966-019-0839-9 (PMC6701122; doi:10.1186/s12966-019-0839-9)
Supplement: Supplementary file 4 — Table S1. Prevalence of physical activity per domain and sitting time among South American countries by sociodemographic characteristics. (DOC 84 kb) [file 12966_2019_839_MOESM4_ESM.doc]

**Additional file 4: Table S1.** Prevalence of physical activity per domain and sitting time among South American countries by sociodemographic characteristics.

|  |  |  |  |  | **Countries** |  |  |
| --- | --- | --- | --- | --- | --- | --- | --- |
|  |  | **Argentina** | **Brazil** | **Chile** | **Ecuador** | **Peru** | **Suriname** |
| **Leisure physical active (≥150min/week)** | | |  |  |  |  |  |
| Sex | Men | 29.2 (27.9 to 30.4) | 22.1 (21.2 to 23.1) | 27.1 (23.7 to 30.9) | 23.6 (22.0 to 25.3) | 12.8 (11.5 to 14.3) | 25.1 (22.9 to 27.5) |
|  | Women | 29.4 (27.5 to 31.3) | 18.7 (17.9 to 19.5) | 14.7 (12.6 to 17.0) | 7.7 (6.8 to 8.6) | 4.7 (4.0 to 5.5) | 10.0 (8.8 to 11.4) |
| Age group | 18-34y | 35.0 (33.3 to 36.7) | 25.0 (23.9 to 26.1) | 29.4 (25.6 to 33.5) | 19.6 (18.2 to 21.0) | 14.7 (13.2 to 16.2) | 24.2 (21.9 to 26.7) |
|  | 35-49y | 27.9 (26.1 to 29.7) | 16.8 (15.9 to 17.8) | 15.0 (12.3 to 18.1) | 11.8 (10.6 to 13.3) | 5.3 (4.3 to 6.6) | 13.5 (11.7 to 15.4) |
|  | 50-64y | 19.9 (18.3 to 21.7) | 16.8 (15.7 to 18.1) | 15.8 (12.4 to 20.0) | 8.5 (6.5 to 11.1) | 2.4 (1.7 to 3.6) | 9.4 (7.7 to 11.5) |
| Educational status | No education | 23.4 (15.5 to 33.8) | 8.6 (6.7 to 10.8) | 28.4 (10.2 to 57.9) | 4.4 (2.3 to 8.0) | 1.3 (0.6 to 3.0) | 4.5 (2.5 to 7.9) |
|  | Less than secondary | 24.1 (22.6 to 25.7) | 13.3 (12.5 to 14.2) | 15.7 (12.3 to 19.9) | 14.4 (13.4 to 15.5) | 5.6 (4.8 to 6.5) | 15.9 (14.3 to 17.5) |
|  | Secondary education | 31.7 (30.0 to 33.4) | 23.9 (22.9 to 25.0) | 22.9 (20.3 to 25.8) | 19.7 (14.9 to 25.6) | 11.9 (10.4 to 13.4) | 26.4 (22.8 to 30.4) |
|  | College or more | 37.2 (34.6 to 39.9) | 32.6 (30.8 to 34.4) | 23.6 (16.6 to 32.3) | 19.0 (16.8 to 21.5) | 9.3 (7.5 to 11.4) | 26.5 (20.8 to 33.0) |
| Wage | Minimum wage | 25.3 (23.3 to 27.4) | 22.2 (21.4 to 23.2) | 14.8 (11.9 to 18.1) | - | - | 13.3 (10.2 to 17.1) |
|  | More than minimum | 30.3 (29.1 to 31.5) | 16.6 (15.0 to 18.3) | 23.0 (20.3 to 25.9) | - | - | 19.0 (16.8 to 21.3) |
| **Transport physical activity (≥10min/week)** | | |  |  |  |  |  |
| Sex | Men | 62.7 (61.2 to 64.3) | 48.5 (47.4 to 49.6) | 68.0 (64.0 to 71.8) | 13.8 (12.5 to 15.3) | 67.9 (65.8 to 69.8) | 27.6 (25.3 to 29.9) |
|  | Women | 65.6 (63.3 to 67.8) | 53.9 (52.9 to 54.9) | 64.5 (61.3 to 67.6) | 4.1 (3.5 to 4.8) | 71.4 (69.8 to 73.1) | 27.5 (26.7 to 29.4) |
| Age group | 18-34y | 64.0 (62.0 to 65.9) | 52.8 (51.6 to 54.0) | 67.3 (63.2 to 71.2) | 9.6 (8.6 to 10.7) | 68.1 (66.0 to 70.1) | 28.3 (25.9 to 30.8) |
|  | 35-49y | 62.2 (60.0 to 64.4) | 51.2 (50.0 to 53.5) | 63.0 (58.4 to 67.4) | 8.0 (7.0 to 9.1) | 70.2 (68.1 to 72.3) | 27.9 (25.6 to 30.3) |
|  | 50-64y | 64.7 (62.1 to 67.1) | 48.9 (47.3 to 50.5) | 69.5 (65.3 to 73.5) | 7.9 (6.2 to 10.0) | 71.8 (69.1 to 74.4) | 25.4 (22.8 to 28.2) |
| Educational status | No education | 75.2 (62.8 to 84.5) | 51.6 (48.1 to 55.1) | 72.7 (51.7 to 86.8) | 10.6 (6.5 to 17.0) | 72.9 (67.6 to 77.5) | 25.0 (20.6 to 30.0) |
|  | Less than secondary | 67.1 (65.0 to 69.1) | 55.5 (54.3 to 56.7) | 67.8 (63.3 to 71.9) | 9.6 (8.7 to 10.7) | 75.1 (73.3 to 76.7) | 28.8 (27.0 to 30.6) |
|  | Secondary education | 63.3 (61.3 to 65.3) | 50.8 (49.6 to 52.0) | 66.8 (63.5 to 69.9) | 7.9 (5.4 to 11.4) | 67.6 (65.3 to 69.7) | 28.5 (24.9 to 32.4) |
|  | College or more | 55.5 (52.6 to 58.4) | 41.5 (39.6 to 43.4) | 56.4 (45.9 to 66.3) | 5.5 (4.4 to 6.8) | 63.3 (59.8 to 66.7) | 17.7 (13.2 to 23.4) |
| Wage | Minimum wage | 69.3 (66.7 to 71.8) | 72.4 (70.6 to 74.2) | 72.2 (68.3 to 75.9) | - | - | 25.6 (21.8 to 29.9) |
|  | More than minimum | 62.1 (60.6 to 63.5) | 50.1 (49.1 to 51.2) | 63.6 (60.3 to 66.8) | - | - | 25.2 (22.8 to 27.7) |
| **Occupational physical activity (≥10min/week)** | | |  |  |  |  |  |
| Sex | Men | - | 27.6 (26.6 to 28.7) | 63.1 (59.2 to 67.0) | - | 66.1 (64.1 to 68.1) | 55.2 (52.7 to 57.7) |
|  | Women | - | 9.8 (9.2 to 10.4) | 57.8 (54.4 to 61.0) | - | 39.7 (38.0 to 41.4) | 48.6 (46.5 to 50.6) |
| Age group | 18-34y | - | 18.7 (17.8 to 19.7) | 56.9 (52.7 to 61.1) | - | 44.5 (42.4 to 46.6) | 53.7 (51.1 to 56.4) |
|  | 35-49y | - | 20.5 (19.5 to 21.6) | 63.2 (58.8 to 67.4) | - | 56.8 (54.6 to 59.0) | 53.7 (51.0 to 56.3) |
|  | 50-64y | - | 14.7 (13.7 to 15.8) | 61.6 (57.0 to 66.1) | - | 53.8 (50.9 to 56.6) | 44.8 (41.7 to 47.8) |
| Educational status | No education | - | 17.8 (15.5 to 20.4) | 59.1 (37.1 to 77.9) | - | 57.8 (52.4 to 63.1) | 44.3 (39.2 to 49.6) |
|  | Less than secondary | - | 24.5 (23.5 to 25.5) | 65.3 (60.8 to 69.5) | - | 57.6 (55.6 to 59.6) | 53.5 (51.5 to 55.5) |
|  | Secondary education | - | 15.9 (15.0 to 16.9) | 62.1 (58.9 to 65.2) | - | 45.0 (42.7 to 47.3) | 54.1 (49.9 to 58.2) |
|  | College or more | - | 8.3 (7.3 to 9.3) | 31.9 (23.7 to 41.3) | - | 49.0 (45.6 to 52.4) | 44.4 (38.0 to 51.0) |
| Wage | Minimum wage | - | 34.1 (32.2 to 36.1) | 60.3 (57.1 to 63.4) | - | - | 45.9 (41.4 to 50.5) |
|  | More than minimum | - | 25.1 (24.2 to 26.1) | 64.1 (59.6 to 68.4) | - | - | 50.0 (47.3 to 52.7) |
| **Sitting time (≥4h/day)** | |  |  |  |  |  |  |
| Sex | Men | 58.3 (56.9 to 59.7) | 12.4 (11.7 to 13.1) | 37.2 (33.3 to 41.3) | - | 78.0 (76.3 to 79.6) | 52.2 (49.6 to 54.7) |
|  | Women | 58.6 (56.6 to 60.7) | 16.9 (16.2 to 17.7) | 33.8 (30.6 to 37.1) | - | 79.5 (78.0 to 81.0) | 53.8 (51.7 to 55.8) |
| Age group | 18-34y | 61.5 (59.7 to 63.2) | 16.4 (15.5 to 17.3) | 43.5 (39.2 to 47.8) | - | 82.8 (81.2 to 84.4) | 54.6 (51.9 to 57.2) |
|  | 35-49y | 56.0 (54.0 to 58.0) | 12.8 (12.1 to 13.7) | 29.7 (25.8 to 33.8) | - | 75.3 (73.4 to 77.2) | 51.4 (48.8 to 54.0) |
|  | 50-64y | 55.8 (53.5 to 58.1) | 14.5 (13.5 to 15.6) | 31.6 (26.8 to 36.8) | - | 77.5 (75.0 to 79.7) | 52.3 (49.2 to 55.3) |
| Educational status | No education | 44.2 (33.0 to 56.0) | 13.2 (11.2 to 15.6) | 22.5 (10.4 to 42.1) | - | 74.4 (69.5 to 78.8) | 36.5 (31.5 to 41.9) |
|  | Less than secondary | 50.5 (48.6 to 52.3) | 15.6 (14.8 to 16.5) | 25.5 (21.6 to 29.7) | - | 74.0 (72.3 to 75.7) | 49.9 (47.9 to 51.9) |
|  | Secondary education | 62.8 (61.0 to 64.6) | 16.1 (15.2 to 17.0) | 37.9 (34.6 to 41.4) | - | 81.6 (79.8 to 82.3) | 62.9 (58.8 to 66.8) |
|  | College or more | 69.8 (67.3 to 72.2) | 9.4 (8.4 to 10.6) | 55.8 (45.6 to 65.6) | - | 83.1 (80.4 to 85.5) | 73.2 (67.1 to 78.5) |
| Wage | Minimum wage | 50.8 (48.5 to 53.2) | 13.0 (11.7 to 14.4) | 23.5 (19.8 to 27.7) | - | - | 44.6 (40.2 to 49.0) |
|  | More than minimum | 60.5 (59.2 to 61.8) | 10.6 (9.9 to 11.2) | 40.0 (36.8 to 43.3) | - | - | 58.0 (55.3 to 60.6) |

Note. Values are presented in percentage and 95% confidence intervals. Y=years.
